# Autosomal dominant transmission of transient neonatal lactic acidosis: a case report

**DOI:** 10.1186/s12887-020-02085-x

**Published:** 2020-04-20

**Authors:** Emily B. Mardian, Matthew A. Lines, Care4Rare Consortium, Gregory P. Moore

**Affiliations:** 1 Faculty of Medicine, University of Ottawa, Ottawa, Ontario Canada; 2 Division of Metabolics and Newborn Screening, Department of Pediatrics, Children’s Hospital of Eastern Ontario, Ottawa, Ontario Canada; 3 Children’s Hospital of Eastern Ontario Research Institute, Ottawa, Ontario Canada; 4 Division of Neonatology, Department of Pediatrics, Children’s Hospital of Eastern Ontario, Ottawa, Ontario Canada; 5 Division of Newborn Care, Department of Obstetrics and Gynaecology, The Ottawa Hospital-General Campus, Box 806, 401 Smyth Road, Ottawa, Ontario K1H 8L1 Canada

**Keywords:** Primary lactic acidosis, Transient lactic acidosis, Inborn errors of metabolism, Neonate

## Abstract

**Background:**

Lactic acidosis is a common finding in neonates, in whom mitochondrial dysfunction is often secondary to tissue hypoperfusion, respiratory failure, and/or sepsis. Primary (non-physiological) lactic acidosis is comparatively rare, and suggests the presence of an inborn error of mitochondrial energy metabolism. Optimal medical management and accurate prognostication requires the correct determination of the etiology of lactic acidosis in a given patient. Unfortunately, genetic diagnoses are rare and highly variable for neonates presenting with primary lactic acidosis; individual case reports may offer the most promise for treatment considerations.

The mitochondrion is a complex molecular machine incorporating the products of > 1000 distinct nuclear genes. Primary lactic acidoses are therefore characterized by high genetic heterogeneity and a specific genetic diagnosis currently remains out of reach in most cases. Most mitochondriopathies with neonatal onset follow autosomal recessive inheritance and carry a poor prognosis. Here we detail the case of a father and daughter with dominantly-inherited, resolving (i.e. transient) neonatal hyperlactatemia due to complex IV deficiency. We found no other published descriptions of benign transient complex IV deficiency with autosomal dominant inheritance.

**Case presentation:**

Both individuals presented as neonates with unexplained, marked lactic acidosis suggesting a primary mitochondrial disorder. Within the first weeks of life, elevated blood lactate levels normalized. Their clinical and developmental outcomes were normal. Biochemical studies in the proband showed multiple abnormalities consistent with a complex IV respiratory chain defect. Cultured skin fibroblasts showed an elevated lactate-to-pyruvate ratio, deficient complex IV activity, and normal pyruvate dehydrogenase and pyruvate carboxylase activities. Whole-exome sequencing of the proband and both parents did not identify a causative mutation.

**Conclusion:**

We conclude that the proband and her father appear to have a dominant form of transient neonatal hyperlactatemia due to heterozygous changes in an as-yet unidentified gene. This transient neonatal complex IV deficiency should be considered in the differential diagnosis of primary neonatal hyperlactatemia; notable clinical features include autosomal-dominant inheritance and an apparently benign postnatal course. This report exemplifies the growing differential diagnosis for neonatal lactic acidosis and highlights the importance of both physician counselling and the use of family history in communicating with parents.

## Background

Lactic acid is an anaerobic metabolite that tends to accumulate whenever cellular respiration is inadequate to meet cellular energy requirements. Commonly, lactic acidosis reflects acquired mitochondrial dysfunction due to impaired tissue oxygenation (‘shock’). In neonates, hyperlactatemia is commonly observed in sepsis, hypoxic-ischemic injury, and congenital heart disease. When secondary, blood lactate levels mirror the patient’s clinical state, resolving with correction of the underlying disease process [1]. Primary lactic acidosis that is inappropriate to the clinical context suggests an inborn error of cellular energy metabolism. Collectively, these primary lactic acidoses are numerous. They are genetically heterogeneous with disorders of mitochondrial oxidative phosphorylation or pyruvate metabolism accounting for most cases. Because the mitochondrion is complex, comprising the products of > 1000 distinct nuclear genes, genetic diagnosis of primary mitochondrial disorders is rarely found, occurring only in ~ 25–50% of cases [2, 3]. With few exceptions (e.g. holocarboxylase synthetase deficiency, which is treatable with biotin, and pyruvate dehydrogenase deficiency, which is somewhat treatable with the ketogenic diet), specific treatments are lacking, and many patients die in infancy or childhood [4]. Here we detail the case of a father and daughter with dominantly-inherited, resolving (i.e. transient) neonatal hyperlactatemia due to transient complex IV deficiency. No previous description of such an autosomal dominant form of benign, transient complex IV appears to exist in the literature; this reiterates the increasing differential diagnosis for lactic acidosis in a neonate.

## Case presentation

### Ethics approval and consent to participate

Given this was a case report, the Children’s Hospital of Eastern Ontario Research Ethics Board did not need to approve the work, as per their regulations. The parents provided written consent for their daughter’s clinical information to be disseminated as a case report.

### Patient information

A newborn female was born at 40 + 3 weeks gestation (birth weight 3117 g) after a vacuum-assisted delivery secondary to fetal decelerations and failure to progress following an otherwise uncomplicated pregnancy. Apgar scores were 7 and 8 at 1 and 5 min, respectively. She had ongoing mild hypoglycemia and temperature instability over her first 18 h resulting in admission to the Neonatal Intensive Care Unit. She then developed moderate respiratory distress and regurgitation of feeds.

### Clinical findings

Arterial bloodwork at 19 h of life revealed: serum lactate 19.8 mmol/L (Reference interval [RI]: < 2.2 mmol/L), pH 7.26, p_a_CO_2_ 12 mmHg, p_a_O_2_ 131 mmHg, bicarbonate 5 mmol/L, and elevated transaminases (AST 243 U/L, ALT 54 U/L) and alkaline phosphatase (333 U/L). At 24 h of life, thiamine (100 mg every 12 h) and biotin (8 mg every 12 h) were initiated via nasogastric tube and continued for 4 days. Over the following 24 h, the elevated lactate levels, hypoglycemia, and respiratory distress progressively improved with resulting clinical stability. An echocardiogram and serum creatinine kinase were normal. The patient had routine metabolic studies performed at 21 h of life. Plasma amino acids showed several abnormalities including increased alanine (936 μmol/L; RI 131–710), proline (1001 μmol/L; RI 110–417), glutamine (946 μmol/L; RI 376–709), and tyrosine (396 μmol/L; RI 55–147), consistent with mitochondrial and hepatic dysfunction. Urine organic acids showed increased excretion of lactic acid and tyrosine metabolites (the latter being a non-specific indication of liver dysfunction). Acylcarnitine profile showed increased glutarylcarnitine (0.29 μmol/L; RI 0.25) and multiple long chain acylcarnitine species. Muscle biopsy was deferred given the patient’s clinical improvement. Cultured skin fibroblasts exhibited normal pyruvate dehydrogenase and pyruvate carboxylase activities, a markedly elevated lactate:pyruvate ratio (59.66, RI 10–25) and reduced complex IV activity (2.69 nmol/min/mg protein, RI 4–12). The patient was discharged home at 7 days of life after all clinical concerns had resolved several days prior.

### Follow-up

The patient’s clinical and laboratory findings were not in keeping with holocarboyxlase synthetase deficiency, and biotin was discontinued after 4 days of treatment. Thiamine was also simultaneously discontinued with no observable effect on the blood lactate level. The blood lactate level remained only mildly elevated (3–4 mmol/L), as did the AST and ALT prior to discharge. All of these had normalized by follow-up at 6 months of age. At 10 months follow-up, physical examination and blood results were normal. The patient met all expected developmental, gross motor, fine motor, and language milestones.

### Family history

Records were sought regarding the patient’s father, who had a similar presentation 32 years prior. He was born at 39 weeks gestation and weighing 3620 g following an uneventful delivery with Apgars of 9 and 10 at 1 and 5 min, respectively. At 12 h, he presented with respiratory distress following feeding and blood work showed an elevated serum lactate of 4.8 mmol/L, pH 7.34, pCO2 28 mmHg, and bicarbonate 15 mmol/L. Lactate levels normalized after a 36 h fast. Plasma amino acids showed multiple elevations including alanine (648 μmol/L; RI < 345), glycine (433 μmol/L; RI < 248), and ornithine (171 μmol/L; RI < 61). He received a low-fat, high-carbohydrate diet for 4 months. Metabolic testing in cultured skin fibroblasts was normal (detailed records unavailable) and no diagnosis was made.

### Outcomes

The observed father-daughter transmission of the phenotype in this kindred was potentially compatible with autosomal dominant (or, less likely, X-linked dominant) inheritance but not with matrilineal (mtDNA-based) inheritance. To study the genetic etiology of the primary lactic acidosis in this kindred, whole-exome sequencing (WES) of the proband and both parents was performed on a research basis (Care4Rare Consortium) after appropriate consents were obtained. Research by the Care4Rare Consortium was approved by the Children’s Hospital of Eastern Ontario Research Institute Research Ethics Board (ref #11/04E). The genomic analysis did not identify any pathogenic, likely-pathogenic or compelling candidate variant(s) to account for the clinical phenotype in the family. Specifically, no pathogenic or likely-pathogenic variants were detected in *TRMU* (mitochondrial tRNA modifying enzyme).

## Discussion and conclusions

We describe a father and daughter with transient neonatal lactic acidosis, mild transient hepatic dysfunction, spontaneous clinical resolution, and no apparent developmental sequelae. Complex IV activity and lactate:pyruvate ratio were markedly abnormal in skin fibroblasts derived from the daughter. This disorder appears to represent an autosomal dominant (or, less likely, X-linked-dominant) form of transient complex IV deficiency caused by mutation of an as-yet unidentified nuclear gene.

Mitochondrial disorders are among the most common and heterogeneous inherited metabolic diseases in humans, with > 185 known (and several hundred unknown) genetic types [5, 6]. Inheritance is commonly autosomal recessive or maternal, but may be autosomal dominant or X-linked. Although most mitochondrial disorders are permanent (non-remitting), several previous papers do describe reversible infantile respiratory chain defects. They report their patients having neonatal or infantile presentations lasting weeks to months (e.g. one or more of hypotonia, liver failure, prolonged feeding difficulties, or respiratory insufficiency) with or without later sequelae such as feeding intolerance, hypotonia, hepatopathy, respiratory weakness, or motor delays in the context of a persisting myopathy [7–9]. In contrast, the individuals presented in this report had both a relatively benign neonatal presentation and an apparently normal clinical outcome. Complex IV deficiency states are themselves genetically heterogeneous, with at least 20 distinct genetic causes [10]. Most pediatric-onset complex IV disorders are recessive, life-limiting conditions affecting the brain, cardiac muscle, liver, and other essential body systems. Although we are aware of a dominant form of *SCO2* deficiency associated with severe myopia [11], and a matrilineally-inherited benign transient form caused by m.14674 T > C homoplasmy [8], we are not aware of any other published descriptions of benign transient complex IV deficiency with autosomal dominant inheritance.

Although rapidly becoming the first-line diagnostic test of choice for suspected mitochondrial disorders, WES results in a high-probability gene assignment in < 1/3 of individuals tested [12, 13]. This is in accord with the general WES-associated molecular diagnostic rate of 20–30% for other unsolved Mendelian disorders [14, 15]. Potential reasons for the modest molecular diagnostic rate are manifold. At present, we are aware of only 6000 human disease genes, and we suspect that there are many more with estimates of 6000–13,000 disease genes yet to be identified [16]. Furthermore, the variation responsible for the transient lactic acidosis may not be detectable by “exon” level methods such as variation in the non-coding region, a chromosomal deletion or duplication, complex chromosomal rearrangement(s) or expansion mutations. Technological advances such as whole-genome sequencing and RNA-Seq may resolve some of these issues and provide answers for the family in the near future.

The transient disorder described in this report should be considered a rare exception to the poor prognosis characteristic of most primary lactic acidoses [17]. In the absence of an accurate clinical and family history, transient primary hyperlactatemia may easily be mistaken for typical etiologies of lactic acidosis such as sepsis, congenital heart disease, and perinatal hypoxic-ischemic injury. Although uncommon, the condition presented here should be considered when counseling parents of neonates with primary lactic acidosis regarding prognosis. This case report emphasizes how the incorporation of parents, and their family history, may be vital to providing the most comprehensive overview of the neonate’s status and potential prognosis. Physicians also need to have this transient primary lactic acidosis on their differential diagnosis. While providing general supportive treatment for a primary lactic acidosis (e.g. vitamins that act as cofactors and antioxidants directed towards specific signs and symptoms), physicians can cautiously note to parents that their neonate may have a transient benign form of the clinical condition. Future genetic counselling and prenatal advice could prove useful for families as well [17]. Genetic counselling for inborn errors of metabolism varies by etiology, with this case presenting as an apparently dominant form resulting in a 50% chance of recurrence. Limitations for this case report include the need for future research required to elucidate the causative mutation and pathophysiology.

## Data Availability

Not applicable.

## Abbreviations

RI: Reference interval
WES: Whole-exome sequencing
